# A multi-country analysis of transnational tobacco companies’ market share

**DOI:** 10.18332/tid/157090

**Published:** 2023-01-20

**Authors:** Nikita B. Rajani, Jule Hoelscher, Anthony A. Laverty, Filippos T. Filippidis

**Affiliations:** 1 Department of Primary Care and Public Health, School of Public Health, Imperial College London, London, United Kingdom

**Keywords:** tobacco control, transnational tobacco companies, market share, market trends

## Abstract

**INTRODUCTION:**

The international tobacco market is dominated by five transnational tobacco companies (TTCs) which continue to interfere with measures to reduce tobacco consumption. The aim of this study is to better understand the current international tobacco industry market structure by providing an overview of the market share of these five companies globally.

**METHODS:**

A longitudinal multi-country study design was used to understand market share trends across 90 different countries from 2011 to 2020. Descriptive analyses were conducted based on market share and market size data obtained from Euromonitor Passport. Market share (%), maximal market share (%) and cumulative market share (%) were calculated. Maps and boxplots are used to present the descriptive analyses. Median cumulative TTC market share and interquartile ranges for each year were calculated and stratified by country income level.

**RESULTS:**

The average maximal market share of one company in a country was 50% (IQR: 40.0–63.5) in 2020 compared to 51.5% in 2011 (IQR: 41.3–69.0). One of the five TTCs had the highest market share in 77 out of the 90 countries. Philip Morris International was the main market player in 38 countries, followed by British American Tobacco (24), Japan Tobacco International (8), Imperial Brands (6), and lastly China National Tobacco Corporation was only dominant in China. The percentage of cigarettes manufactured by one of the five TTCs remained relatively stable between 2011 (86.4%) and 2020 (85.2%). Average cumulative TTC market shares increased between 2011 and 2020 in both low- and middle-, and high-income countries.

**CONCLUSIONS:**

The international tobacco market is concentrated with a small number of large players, and this has not changed substantially between 2011 and 2020. The impact of this on the ability of the tobacco industry to resist policy changes is unknown but presents a cause for concern.

## INTRODUCTION

Although significant progress in combatting the tobacco epidemic has been achieved, consumption of tobacco remains a major preventable cause of mortality and disability^1^. Recent statistics show that tobacco is responsible for approximately 8 million deaths and a cost of US$ 1.4 trillion to the global economy annually^1^. The Framework Convention on Tobacco Control (FCTC), introduced by the World Health Organization (WHO) in 2005, set policies to reduce both demand and supply of tobacco products. To provide further practical guidance to countries in implementing the FCTC, the WHO introduced the MPOWER tool. MPOWER advises countries to: monitor tobacco use (M), protect people from tobacco smoke (P), offer methods to quit tobacco use (O), warn about the dangers of tobacco (W), enforce bans on promotion and advertisements (E), and raise taxes to reduce consumption (R)^2^.

The number of countries adopting MPOWER strategies continues to increase every year with almost 70% of the global population being covered by at least one MPOWER measure^1^. The adoption of at least one highest-level MPOWER measure among 43 nations was found to be associated with the aversion of 14.6 million smoking attributed deaths between 2014 and 2016^3^. Despite the substantial impact of implementing MPOWER strategies and global efforts to reduce tobacco consumption, significant policy implementation challenges remain with research showing that almost a quarter of the world’s countries have not yet adopted a single MPOWER measure at the best practice level^1^.

One of these challenges is the interference of the tobacco industry which continues to hinder the effective implementation of tobacco control measures and negatively influences policymaking. Five companies, referred to collectively as Transnational Tobacco Companies (TTCs), dominate the international tobacco market. The TTCs consist of British American Tobacco (BAT), Japan Tobacco International (JTI), Philips Morris International (PMI), Imperial Brands (IMB), and China National Tobacco Corporation (CNTC)^4^. Some of the identified tactics adopted by TTCs to ‘prevent, delay or divert tobacco control policies’ include smuggling and illicit trade, litigation and lobbying, disputing and suppressing public health information, and influencing trade treaties to make approval of public health regulations more difficult^1,5-9^. The dominance and global scale of the TTCs provides them with the political and financial resources to circumvent and lessen the impact of tobacco control policies^6^.

Consequently, it is important for researchers and policymakers to be aware of trends and changes in competitive structures and respective market shares of TTCs in both global and national markets. Some previous studies have focused on the competitive structure of the tobacco industry and TTCs’ strategies to deal with tobacco control policy, but with a singular country or region as the focus rather than at a global level^7-9^. The aim of this study is to contribute to a better understanding of the current international tobacco industry market structure by providing an overview of developments in TTC market shares globally. Increased awareness and a higher level of transparency can help policymakers, researchers and regulatory agencies better understand TTC presence in the international tobacco market and help inform the development of policies to combat tobacco industry interference.

## METHODS

### Data sources and measures

A longitudinal multi-country study design was used to understand market share trends of TTCs across 90 different countries. Descriptive analyses were conducted based on market share and market size data obtained from Euromonitor Passport, an online database of a market research company called Euromonitor International^10^. Euromonitor International is a provider of strategic market research across various industries and countries. Euromonitor Passport collects annual data based on a combination of official national statistics and unofficial sources such as company reports and media articles^10^. Data from 90 countries across 10 years (2011–2020) were available in Euromonitor Passport.

Retail volume of cigarettes defined by ‘millions of cigarette sticks sold’ was used to determine market size. Market share for each TTC was operationalized using ‘percentage of sticks of cigarettes’ from the Euromonitor Passport database. Cigarette sticks did not include roll-your-own or hand-rolled cigarettes. Maximal market share was defined as the highest market share by cigarette retail volume held by a single company in one country and was extracted using relevant country-level data for each TTC. For each year from 2011 to 2020, cumulative TTCs market share was calculated by adding market shares of the five TTCs. Since Imperial Tobacco Group was renamed in 2016 to IMB, data from 2011 to 2015 are for Imperial Tobacco Group and data from 2016 to 2020 are for IMB^11^.

Countries were classified into high income countries (HICs) and low- and middle-income countries (LMICs). These categories were based on the World Bank classification which uses gross national income per capita to classify countries^12^. Countries were also categorized into the six WHO regions: African Region (AFR), Regions of the Americas (AMR), Eastern Mediterranean Region (EMR), European Region (EUR), South-East Asian Region (SEAR), and Western Pacific Region (WPR)^13^.

### Statistical analysis

Before analyses were conducted, changes in market shares of more than 20% for one company within one year (n=5) were verified against reports of company acquisitions by others and removed if no explanation for the change was found (n=1)^14-17^. Missing data points were also excluded. Descriptive analyses were conducted to calculate market share, maximal market share of a tobacco company in each country, and cumulative market share which was the total market share of all five TTCs globally. Median maximal market shares and interquartile ranges (IQR) for each year were also calculated and presented in the Supplementary file tables. The online software, MapChart was used to create maps presenting market share data across different countries^18^. The descriptive analyses were further stratified for HICs (n=39) and LMICs (n=51).

## RESULTS

**Figure 1:**
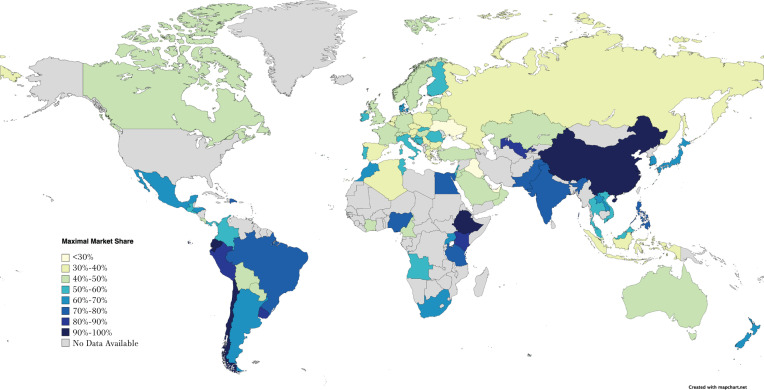
Map showing maximal market share percentage held by a single company in 2020 in 90 countries

Figure 1 displays the distribution of the maximal market share percentage held by a single company in one country in the year 2020. The map shows that across the 90 countries, there was a large variation in maximal market share of TTCs with approximately 22% maximal market share in Iraq and up to 100% maximal market share in Ethiopia and Ecuador. Among the WHO regions, maximal market shares in 2020 were lowest in the European region (median: 43.0%; IQR: 38.0–50.0) and highest in the Western Pacific Region (62.5%, IQR: 51.8–66.8). The median maximal market share of TTCs in all countries in 2020 was 50.0% (IQR: 40.0–63.5) compared to 51.5% in 2011 (IQR: 41.3–69.0) (Supplementary file Table 1). Median maximal market share of TTCs in HICs was 45.0% (IQR: 39.5–54.5) in 2011 and 44.0% (IQR: 38.5–54.5) in 2020. For LMICs, median maximal market share was 60.0% (IQR: 46.0–75.0) in 2011 and 57.0% (IQR: 43.0–71.0) in 2020 (Supplementary file Table 1).

**Figure 2:**
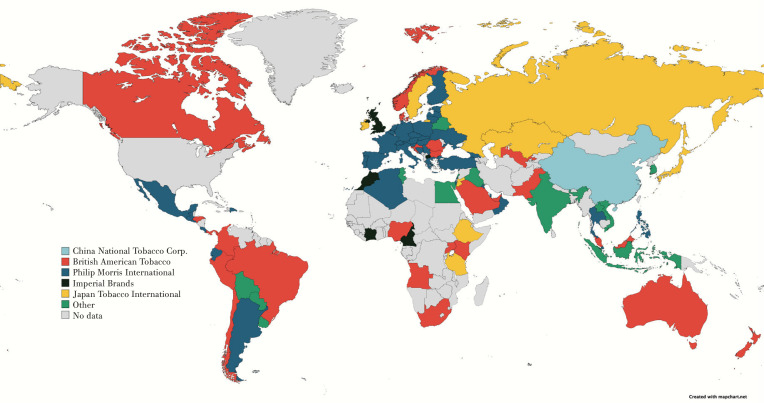
Map showing the company with the highest market share of cigarettes by retail volume in 90 countries

Figure 2 illustrates the TTCs with the highest market share percentage in each of the 90 countries in the analysis. In 77 out of the 90 countries, one of the five TTCs was the company with the highest share in the market, and in 13 of the 90 countries a company other than one of the five TTCs was the dominant market player. PMI was the main market player in 38 of the countries, followed by BAT which held the highest market share in 24 countries, JTI in 8 countries, IMB in 6 countries, and lastly CNTC was only dominant in China. PMI was particularly dominant in the European region (EUR) whilst BAT plays a key role in the tobacco market in the region of the Americas (AMR). Based on the analyzed data, approximately 86.4% of cigarettes sold in 2020 across the 90 countries assessed were manufactured by one of the five TTCs compared to 85.2% in 2011 (Supplementary file Table 2). The company with the largest market share from the five TTCs was CNTC which produced 52.0% of the cigarettes sold in 2020. This is an absolute increase of 6.8% compared to the market share of CNTC in 2011. After excluding the Chinese cigarette market, PMI which was also dominant in the greatest number of countries, had the highest market share of 27.7% followed by BAT with 21.4% market share.

**Figure 3:**
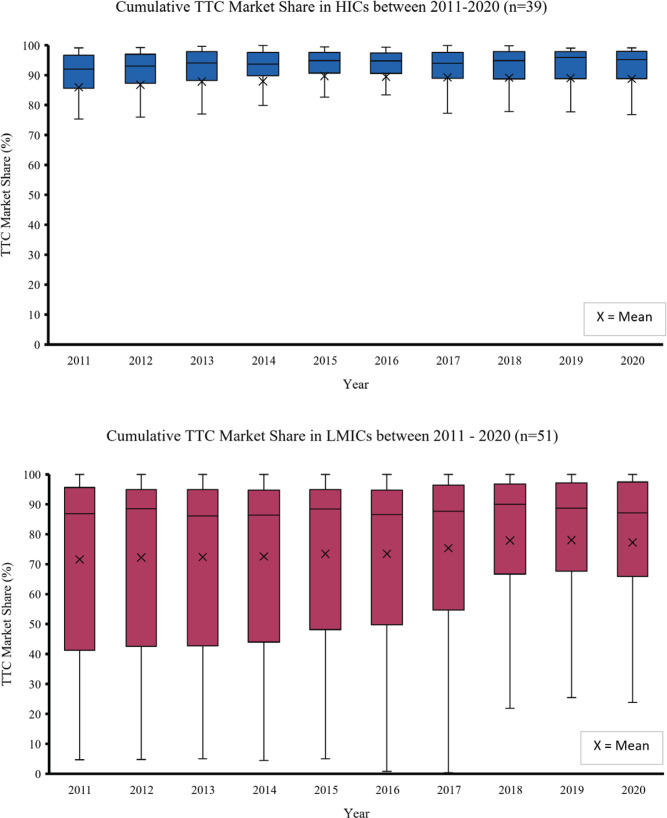
Cumulative market shares of the five TTCs combined in HICs (blue, n= 39) and LMICs (red, n=51), 2011–2020

The upper boxplot (blue) in Figure 3 shows the cumulative market share of TTCs in 39 HICs between 2011 and 2020 and the lower boxplot (red) shows the cumulative market share of TTCs in 51 LMICs. In 2020, the median cumulative market share of TTCs was higher in HICs (95%; IQR: 89–98) compared to LMICs (87%; IQR: 68–97). Mean cumulative TTC market share increased in HICs from 86.0% in 2011 to 88.8% in 2020. Similarly, cumulative TTC market share increased in LMICs from 71.7% to 77.3%; however, a higher level of variation is evident in LMICs compared to HICs.

## DISCUSSION

Our analysis shows that TTCs control a large share of the tobacco market in the majority of the 90 countries we assessed, with more variation in LMICs, where the cumulative market share of TTCs was lower than in HICs. We also found that, on average, one company controlled approximately 50% of the national market, and the percentage of manufactured cigarettes from one of the five TTCs had not substantially changed. Although high variation can be seen when comparing individual countries, the median maximal market share was found to have marginally decreased between 2011 and 2020.

Several developments and factors can be associated with high TTC market shares and increased competition between players in the tobacco market^19,20^. For example, trade liberalization has allowed foreign companies to introduce new products onto the market, fostering higher levels of competition^20^. Similarly, the privatization of state-owned companies over the last decade is also seen as a factor contributing to higher levels of competition and increased cigarette sales, which could have possibly played a role in strengthening the position of the TTCs^19,20^. Higher competition is likely to have fueled marketing activity and resulted in an increase in the respective market shares of individual TTCs whilst lowering the level of dominance of one single company^19^. China is the obvious exception with the state-owned CNTC dominating the largest cigarette market in the world. Although CNTC operates primarily in China and has not always been given the same attention as the other TTCs, it is increasingly viewed as a company mimicking the globalization strategies of TTCs, which should make it a key target of tobacco control efforts^21^.

A key finding of our analysis was that one of the five TTCs was dominant in 77 out of the 90 countries analyzed. There were only 13 countries where one of the TTCs did not hold the highest market share; this demonstrates the concentration of the tobacco industry market by a few large companies. PMI controlled the market in many European countries whilst BAT held leading shares in the region of the Americas. The dominance of these five companies creates an international oligopolistic market which is likely to persist due to the high entry barriers for new companies and scale advantages of the dominant incumbents. With higher taxes, bans and regulations on the tobacco market and the structural power of TTCs, it is more difficult for new companies to enter tobacco markets^19,20^. Holden and Lee^22^ describe the extensive demand creation efforts of TTCs in the form of mass advertising and reinforcement of brand image and consumer loyalty as ‘crucial sources of high concentration in the industry’. The oligopolistic nature of the tobacco industry market allows TTCs to leverage their resources and agency power to undercut new entrants and eliminate competition to ensure oligopolistic profits remain high^22^. According to Hawkins et al.^23^, this type of market structure has allowed TTCs to ‘resist regulation and shape policy environments through lobbying, financial contributions, research funding and formation of front groups’ including curtailing packaging restrictions in countries such as Australia^23,24^. The coordinated action of TTCs at a global level allows them to take advantage of governance structures, challenge policies, delay policy adoption and implementation and overall increase the cost of any new novel tobacco control policies for governments^23^.

We also found that the cumulative share of all TTCs in 2020 was higher in HICs compared to LMICs. This is most likely attributed to the earlier market entry of TTCs in HICs compared to LMICs^4,8^. Regardless, the cumulative share of all five TTCs combined was found to have increased slightly in HICs but generally remained high and stable with little heterogeneity over the last 10 years. This indicates that the presence of the TTCs in the tobacco market of HICs is mature and well-established. Among LMICs, our findings indicate a more upward trend in the cumulative share of TTCs with greater heterogeneity. The high level of variation could be due to tobacco markets in countries such as India, Egypt, Vietnam and Indonesia, not being dominated by a TTC. In many of these countries, the markets are monopolized by national or state-owned companies and are regulated through more stringent trade policies. For example, the tobacco market in Vietnam is dominated by the state-owned Vietnam National Tobacco Corporation which has a strong economic presence preserved by closed trade policies^20^. Such types of markets in Africa and South-East Asia are difficult to penetrate but can be promising prospects for TTCs, causing them to increasingly become their target of expansion^8^. To prevent their dominance in such emerging markets, policymakers in LMICs need to build strong institutions that will enforce tobacco control policies to protect public health^8^. Tobacco control policymakers can learn from the already identified tactics and strategies used by TTCs to successfully penetrate and establish their position in both LMICs and HICs^6-9^.

Research shows that TTCs are using aggressive advertising and competitive pricing strategies to outpace local tobacco companies and establish their presence in the market^8,19,25^. For example, in Taiwan, market shares of the state-owned company, Taiwan Tobacco and Liquor Corporation, are continuing to decrease whilst the market shares of both JTI and BAT are found to be growing^24^. According to Gao et al.^26^, TTCs in Taiwan were able to outperform the state-owned company due to the lack of fiscal pressure from the Taiwanese government which allowed for more complex price segmentation by the four TTCs, which may have colluded on the most convenient price for their popular premium brands. Similarly, Thailand is another example of a country where the market control of a state-owned company, Thailand Tobacco Monopoly, which had exclusive rights to sell cigarettes in Thailand for 60 years, was slowly outperformed by PMI^7^. Using aggressive product placement strategies, political influence and countering existing advertising bans and regulations, PMI was steadily able to increase its market share from 0% in 1991 up to 50% in 2019 when it overtook Thailand Tobacco Monopoly’s market share of 43% and the 7% market share of the other TTCs combined^7,27^.

The steady growth of TTC presence in LMICs and their continuing dominance in HICs remain major public health concerns. The past experience of HICs and countries such as Thailand and Taiwan, can provide guidance to governments, health professionals and tobacco control policymakers in LMICs on what to expect and how to combat opposition or interference from TTCs^4^. Further research on understanding the strategies of TTCs as well as the countermeasures adopted by countries against the tobacco industry could help inform the development of tobacco control policies and support the implementation of the FCTC in countries where TTCs have not yet saturated the market.

### Strengths and limitations

This study uses commercial data for 90 countries from various geographical regions. To the authors’ knowledge, it is the first multi-country analysis that explores the market shares of TTCs and their development over the last decade. The findings of this study provide valuable insights that can guide future research and inform the development and evaluation of tobacco control policies and regulatory decisions. However, further research on the sales and market control of specific cigarette brands across and within the five TTC companies, as well as in specific markets, could provide insights on the behavior and preferences of smokers^26^ and the strategic approaches of TTCs. This could be particularly useful for the development of effective tobacco legislation and strengthen the implementation of FCTC policies.

Although we analyzed data from 90 countries, the available data was skewed towards European countries and HICs. The analysis included data on two-thirds of all HICs globally but only five percent of low-income countries (LICs). The political, financial and social contexts may differ substantially between high- and low-income countries; hence, the findings should be interpreted with this in mind and may not fully reflect the global tobacco market^4^. Moreover, whilst a multi-country analysis spanning over the last 10 years can provide a good snapshot of the current situation and how it has developed, individual country-level cases studies are required to gain a better understanding of how countries are able or in many cases not able to prevent TTCs from gaining a strong foothold in the market. Additionally, although Euromonitor International is a valuable source for market data in tobacco research^24,28^, it is important to acknowledge that Euromonitor International began accepting funds from PMI in 2019^29^, which could have some influence on the data obtained from 2019 onwards.

The market share of TTCs was measured based on retail volume of cigarettes; the production and sale of roll-your-own or hand-rolled cigarettes, and other forms of tobacco, such as bidis or kreteks, have not been accounted for in the analyses. This means that TTCs may exert more dominance than our analysis suggests, particularly in countries where TTCs also control parts of the market for other forms of tobacco (e.g. kretek market in Indonesia)^8^. Since the cigarette market does not exist in isolation and regulatory decisions are not solely based on the key players of the cigarette market, it is important for future research to consider other forms of tobacco and whether they influence TTC dominance and their strategies to interfere with effective tobacco control legislation. Our data also did not consider companies that may have been partially owned by TTCs, which could also have led to a slight underestimation of the overall market shares of TTCs.

Finally, it is also important to note that market share was assessed based on volume rather than value. Under the assumption that the average price of TTC branded cigarettes is higher than of cigarettes manufactured and sold by local companies^30^, it is possible that the market power of the TTCs has been understated. TTCs may aim to increase value rather than volume, at least in certain markets^19^. Conducting value-based analyses to better understand TTC market control would allow for further investigation on the dominance of TTCs; policymakers need to consider how assessing market volume or market value can influence their conclusions with regard to TTCs marketing and pricing strategies.

## CONCLUSIONS

The international tobacco market remains concentrated with a few big companies whose market shares have not largely changed over the last years. Whilst the overall control of TTCs in low- and middle-income countries was found to be lower and much more varied compared to high-income countries, the continuing dominance of TTCs on a global level is concerning because of their large scale and resulting level of resources that allow them to hinder tobacco control efforts. Further research on the sales and market control of specific cigarette brands could provide valuable and in-depth insights on the strategic approaches of TTCs that plan to target and expand their presence in less saturated markets.

## Data Availability

The data supporting this research are available from the authors on reasonable request.
