# Supplementary material for: A multi-country analysis of transnational tobacco companies’ market share
Source: Tob Induc Dis. 2023 Jan 20;21:03. doi: 10.18332/tid/157090 (PMC9853956; doi:10.18332/tid/157090)
Supplement: Supplementary file 1 [file TID-21-03-s1.pdf]

Appendix: Supplementary Tables

Supplementary Table 1. Median maximal market share and interquartile range (IQR) for HICs (n=39), LICs (n=51) and both combined (n=90) from 2011 to 2020

		Median maximal market share (%) in HICs[a], LICs[b] and both combined									
		2011	2012	2013	2014	2015	2016	2017	2018	2019	2020
**HICs**	Median	45.00	46.00	46.00	47.00	45.00	44.00	43.00	46.00	44.00	44.00
	Lower IQR[c]	39.50	40.00	39.50	40.00	40.00	40.00	39.50	40.00	39.00	38.50
	Upper IQR	54.50	55.00	54.50	55.50	55.00	53.50	54.00	54.00	54.00	54.50
**LMICs**	Median	60.00	60.00	61.00	61.00	60.00	57.00	57.00	57.00	54.00	57.00
	Lower IQR	46.00	45.00	43.00	44.50	44.50	44.50	45.00	46.00	44.50	43.00
	Upper IQR	75.00	73.50	73.00	72.00	72.00	71.00	70.00	70.50	70.50	71.00
**Overall**	Median	51.50	51.50	51.50	51.50	51.50	51.50	51.00	50.50	51.00	50.00
	Lower IQR	41.25	41.25	41.25	41.25	41.00	41.25	42.00	41.00	41.00	40.00
	Upper IQR	69.00	68.75	69.00	67.00	65.75	64.75	66.00	67.00	64.75	63.50

[a]*HICs: High-income countries*
[b]*LMICs: Lower-middle income countries*
[c]*IQR: Interquartile range*

Supplementary Table 2. Percentage of overall TTC market share between 2011 and 2020

	Percentage of market share for each TTC and in total from 2011 to 2020 (Including Chinese Market)									
	2011	2012	2013	2014	2015	2016	2017	2018	2019	2020
**BAT**[a]	10.72	10.52	10.30	9.95	10.19	10.50	10.50	10.39	10.38	10.16
**CNTC**[b]	45.27	45.99	46.72	48.00	48.04	47.15	48.56	49.50	49.91	52.03
**IMB**[c]	4.24	4.00	3.78	3.63	3.51	3.38	3.31	3.18	3.19	3.14
**JTI**[d]	8.60	8.62	8.47	8.04	8.09	8.27	8.35	8.44	8.23	7.90
**PMI**[e]	16.39	16.24	15.62	15.42	15.48	15.46	14.98	14.77	14.18	13.16
**Total**	85.23	85.36	84.89	85.03	85.31	84.76	85.71	86.28	85.89	86.40
	**Percentage of market share for each TTC and in total from 2011 to 2020 (Excluding Chinese Market)**									
**BAT**[a]	19.92	19.81	19.86	19.68	20.17	20.42	20.81	20.98	21.15	21.41
**IMB**[c]	7.88	7.53	7.28	7.18	6.94	6.57	6.56	6.42	6.50	6.61
**JTI**[d]	15.82	16.23	16.34	15.91	16.03	16.08	16.55	17.05	16.76	16.65
**PMI**[e]	30.45	30.59	30.13	30.51	30.66	30.07	29.68	29.84	28.87	27.73
**Total**	74.08	74.16	73.61	73.28	73.81	73.51	73.60	74.29	73.28	72.39

[a]*BAT: British American Tobacco*
[b]*CNTN: China National Tobacco Corporation*
[c]*IMB: Imperial Brands*
[d]*JTI: Japan International Tobacco*
[e]*PMI: Philip Morris International*

© 2023 Rajani N.B. et al.
